# Construct-Oriented or Goal-Motivated? Interpreting Test Preparation of a High-Stakes Writing Test From the Perspective of Expectancy-Value Theory

**DOI:** 10.3389/fpsyg.2022.846413

**Published:** 2022-03-07

**Authors:** Shasha Xu

**Affiliations:** School of Foreign Languages, Zhejiang University of Finance & Economics, Hangzhou, China

**Keywords:** test preparation, expectancy-value theory, structural equation modeling, writing assessment, Graduate School Entrance English Examination (GSEEE)

## Abstract

Of the many possible individual factors bearing on test preparation, one is how individuals’ motivational and cognitive perceptions affect test-driven preparation practices. This study reports an investigation into test preparation of a high-stakes writing test from the perspective of expectancy-value theory. Undergraduate students (*n* = 623) on their test preparation for the writing tasks of China’s Graduate School Entrance English Examination (GSEEE) were recruited voluntarily from 11 universities in mainland China. The perceptions of GSEEE test takers, which included goal, task value, task demand, and expectation of success, were identified. Five types of preparation practices were identified for the GSEEE writing tasks: memorizing practice, test familiarization, comprehensive learning, skills development and drilling practice. Structural equation modeling revealed that the expectancy-value model held up well for the paths from test takers’ perceptions to test-driven preparation practices, which were not construct-oriented but goal-motivated. The GSEEE test takers’ goal, determined by the high-stakes nature of admission test, explained their motivation and determined their behavior toward test preparation. Results also indicated that task demand was inadequate to be termed a strong factor in affecting test preparation. As such, the findings of this study offer evidence regarding how an expectancy-value model fit into test preparation mechanism and provide insights into the nature and scope of test preparation for high-stakes writing tests.

## Introduction

High-stakes tests are those tests that have serious consequences attached to them and affect stakeholders to varying degrees (Madaus, 1988; Shohamy, 2007). Access to higher education programs is determined by admission test scores in many countries, including in China, the context of this study. Given the highly competitive education system, high stakes are often associated with admission tests. The influence of tests on teaching and learning has come be known as washback in the field of social sciences such as applied linguistics (Alderson and Wall, 1993). Stakeholders (students and teachers in particular) are inclined to tailor their self-regulated learning and instructional practices to address high-stakes tests. Thus, high-stakes tests carry remarkable implications for different stakeholders, test takers in particular.

Tests of serious consequences for individual stakeholders promote and trigger intensive test preparation and shape preparation practices (Alderson and Hamp-Lyons, 1996; Gulek, 2003; Kiany et al., 2013). Test preparation is conceptualized as an overt dimension of washback on learning (Prodromou, 1995) whereas test preparation for a specific task is defined as a specific dimension of washback (Watanabe, 2004). Compared with washback, with its subtle relation between teaching, learning and assessment remaining under debate, test preparation appears to be more test-driven and goal-oriented (Crocker, 2006). Coaching programs offer excessive coaching, and students devote large amounts of time and effort to cramming for high-stakes tests, a phenomenon often lamented and condemned (e.g., Latham, 1877; Popham, 1991). Some educators believe that the origin of cramming is standardized testing, particularly the objective test format (e.g., Benjamin et al., 1984). Objective test formats (e.g., multiple-choice questions) are believed to facilitate the coping mechanism of test preparation and guessing techniques for test-taking. Other educators argue that the misuse or abuse of test results, rather than test design and format, leads to cramming and triggers negative washback (Yang and Gui, 2007). The discussion of the nature of test preparation is often overwhelmed by the criticism of cramming, which lacks empirical evidence. Further to this, there has been relatively little research on the effects of the subjective test format (e.g., writing tasks) on language learning. The exact path from test takers’ perceptions of test design to their self-regulated learning processes and the nature of their coping mechanisms remains unclear.

Based on the works of Wigfield and Eccles (2000, 2002), students’ self-efficacy beliefs and self-regulated learning strategies have been widely examined in the existing literature (Loh, 2019, for a review). Most of research has been carried out in different academic achievement and learning contexts, such as science subjects (e.g., Guo et al., 2017), mathematics (e.g., Brisson et al., 2017), and language learning (e.g., MacIntyre and Blackie, 2012). The last decade has witnessed the growing interest on motivation and self-regulated learning in English as a foreign language (EFL) context (e.g., Liem et al., 2008; Kim et al., 2015; Bai and Wang, 2020; Gan, 2020; Wang and Gan, 2021). In EFL writing, researchers have explored the nexus of self-efficacy and conceptualized writing self-efficacy (Teng and Zhang, 2016; Teng et al., 2018; Teng, 2021). Despite the growing knowledge of the above constructs, there is a scarcity of research that explores expectancy-value theory in high-stakes assessment context (e.g., Xie and Andrews, 2013; Knekta and Eklöf, 2015; Xu, 2017). The study by Xie and Andrews (2013) was the first attempt to apply expectancy-value theory in the assessment context of the College English Test (CET) in China. In Sweden, Knekta and Eklöf (2015) examined test-taking motivation among Grade 9 students taking a low-stakes or a high-stakes science test and proposed an adapted expectancy-value model in the test situation. Based on Knekta and Eklöf’ (2015) model, Xu (2017) explored the structural relation between expectancy, importance, interest, and test anxiety in the CET listening test. The expectancy-value model by Wigfield and Eccles was adopted in the above three studies with particular reference to four major constructs at the right side of the unabridged model: namely goals and general self-schemata, expectation of success, subjective task value, and achievement-related choices. These studies enlightened the present study to explore the test preparation of the Graduate School Entrance English Examination (GSEEE) from expectancy-value perspective and further investigate potential paths of influence from motivational and cognitive perceptions to test-driven preparation practices.

Test takers have their own stories regarding test preparation and test-taking experiences. In China, English language teaching, learning and testing have retained some of their essentially Chinese features which is characterized by scale and enthusiasm (Jin and Cortazzi, 2002; Cheng and Curtis, 2010). It is against the backdrop of the Chinese higher education system that the present study was conducted, seeking to better understand Chinese university students’ test preparation for a high-stakes writing test. The GSEEE is a large-scale standardized English test at the national level for admissions purposes. It is assumed that the motivational and cognitive perceptions of the test takers have the potential to facilitate the intensity and variety of preparation practices. As a step in this direction, the present study attempts to apply expectancy-value theory to investigate the potential path from test takers’ perceptions to test-driven preparation practices aimed at the GSEEE writing tasks.

## Literature Review

### Expectancy-Value Theory

Among contemporary motivational theories, expectancy-value theory has been examined and applied to explore the relation between motivation and learning. Atkinson (1957, 1964) put forward the original expectancy-value model to link individual achievement behaviors to expectancy-related and task value beliefs. Based on his seminal work, modern expectancy-value theory was expanded to include an array of social and cognitive elements and was tested in educational settings (Eccles et al., 1983; Wigfield, 1994; Wigfield and Eccles, 2000; Flake et al., 2015). Researchers adopting this social cognitive perspective on motivation posit that individuals’ expectation of how well they will do on the activity and the extent to which they value the activity are important determinants of their choices, persistence, and performance. The unabridged expectancy-value model is presented in Wigfield and Eccles (2000, p. 69) to provide the entire scope. Most empirical studies applying this model focus on four major constructs in the four blocks at the right side of the unabridged expectancy-value model: specifically, goals and general self-schemata, expectation of success, subjective task value, and achievement-related choices. Expectation of success and subjective task value are assumed to directly affect achievement-related choices. Expectation of success and subjective task value themselves are assumed to be affected by general self-schemata such as task-specific beliefs, short-term and long-term goals, ideal self, and perceptions of task demands.

A wealth of literature is available on academic motivation research related to expectancy-value theory and motivation-related constructs in different domains such as sports, science, math, and reading (Jacobs and Eccles, 2000; Pajares, 2008; Wigfield and Cambria, 2010; Wan, 2021). There is clear evidence that students’ expectation of success is a strong psychological predictor of their choices, persistence, and performance (e.g., Bong, 2001; Wigfield and Cambria, 2010). Students’ subjective task value predicts both intentions and actual decisions to persist at different activities. Pintrich (1999, 2003) reported the relations among self-regulated learning, task value beliefs and self-efficacy in university and middle school contexts. In the context of colleges/universities, the relation between task value and self-regulated learning across different studies fell into the range of 0.03–0.67 (Pintrich, 1999, p. 463). These empirical studies test theoretically derived hypotheses regarding the relations among motivation-related constructs and quantify their effects on academic achievement.

**FIGURE 1:**
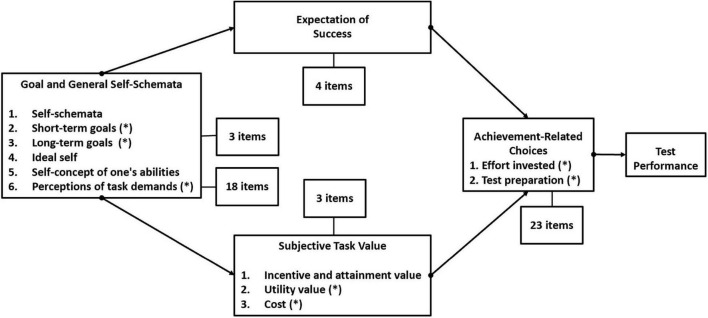
Schematic picture of expectancy-value theory applied to a test preparation context and distribution of questionnaire items.

As noted by Cohen (1998), “preparing for tests and taking them can be an important part of the learning process (Messick, 1982).” Messick defined test preparation as “any intervention procedure specifically undertaken to improve test scores, whether by improving the skills measured by the test or by improving the skills for taking the test, or both” (1982, p. 68). Given its sound explanation in the academic achievement and learning contexts, expectancy-value model is used to conceptualize motivational and cognitive perceptions and achievement-related choices in assessment context (e.g., Xie and Andrews, 2013; Knekta and Eklöf, 2015; Xu, 2017). The simplified and schematic expectancy-value model of Wigfield and Eccles (2000) in a test situation was proposed by Knekta and Eklöf (2015). As a step in this direction, this study proposed an expectancy-value model in test preparation context along with the distribution of questionnaire items (Figure 1). Adopting an expectancy-value perspective on test preparation, the variables investigated in this study include goals and task demand in general self-schemata, expectation of success, utility value and cost in subjective task value, and achievement-related choices.

At the broad conceptual level, the definitions and measures of these constructs in expectancy-value model are extensively discussed (Wigfield and Cambria, 2010, for a review). In the academic setting, long-term and short-term goal refers to the reasons of learners who are engaged in specific learning tasks (Pintrich, 2000; Conley, 2012). Test takers’ short- and long-term goal in this study identifies two types of goal orientation: performance goal, which focuses on the results as compared with others, and mastery goal, which focuses on mastery of tasks. Perception of task demand refers to the skills test takers perceive as necessary for fulfilling the task. Test takers are asked to give their understanding of the task and necessary skill. Task value is perceived as individuals’ desire to perform the task and comprises the incentive and attainment value, utility value and cost (Eccles and Wigfield, 2002; Wigfield and Cambria, 2010). Utility value and cost are used in this study to captures the perceived importance of the task in terms of consequences for the individual. Students are asked various forms of “How importance is it?” to measure task value. Expectation of success is perceived as being closely related to the task to be accomplished (Schunk, 1985; Bandura, 1997), a construct closely related to self-efficacy in language learning (Bandura, 1997). To measure expectation of success, test takers are asked: “Can I do it?” in various forms. Whereas expectancy focuses on one’s expectation of ability, expectation of success emphasizes the believed outcomes (Eccles and Wigfield, 2002) which is relevant to the current test preparation situation.

While the above literature review contributes to the understanding of the expectancy-value model, previous studies indicate several areas of concern for further studies. The first important issue is the dynamic and contextual-situated nature of the key constructs in expectancy-value model (Bandura, 1997; Pajares, 2008). Most studies have focused on the effects of expectation of success and task value on academic achievement and performance. Fewer studies examine their effects on learning or in assessment contexts. Two recent studies (Wan, 2021; Zhan et al., 2021) generated empirical evidence to support the existence of the “expectancy × value” effect in science and language learning, which revealed that the interactions were value-specific and activity-specific. Therefore, it is necessary to characterize and measure the construct of expectation, task value and different types of learning activity in future research. Second, there are ambiguities in the mediating effect of expectation and task value in the theoretical framework (cf. Eccles et al., 1983; Wigfield, 1994; Jacobs and Eccles, 2000). Based on the evaluation of empirical validity of four theoretical models of the relations between achievement goal and expectancy-value theories, Plante et al. (2013) suggest that expectancy-value variables predict achievement-related outcomes both directly and indirectly through achievement goals. Researchers call for more empirical evidence regarding whether a direct path may be established from self-schemata to achievement-related choices (e.g., Plante et al., 2013; Xie and Andrews, 2013; Xu, 2017). There is a need to securitize the paths from motivational and cognitive perceptions to achievement-related choices in a specific context.

### An Expectancy-Value Perspective on Language Assessment Research

With the growing interest in methodological diversity in washback studies, research related to the washback mechanism is no longer confined to qualitative and exploratory approaches. As noted by Qi (2012), emerging research start employing various quantitative approaches to investigate and verify the data in a specific theoretical model, particularly in test preparation studies. Stoneman (2006) investigated students’ test preparation behaviors toward a university exit English test in Hong Kong and concluded that the level of the stakes of a test had different influences on the amount and the type of test preparation. Grounded in the analysis of test preparation for the General English Proficiency Test in Taiwan, Shih (2007) argued that intrinsic, extrinsic and test factors affect the washback in learning and personal psychology. The above studies all contributed to the interpretation of test preparation and, more specifically, to the path from test goals and test characteristics to preparation practices.

Motivation theory in learning psychology is increasingly being adopted by researchers to serve as a theoretical framework to help interpret the mechanism of language learning and test preparation. Chen et al. (2005) surveyed 567 language learners in Taiwan concerning their motivation, expectancy, and self-evaluated skill. Expectancy, strongly related to the required motivation, was found to be an intervening construct between motivation and self-evaluated skill. Chen et al. (2005) concluded that EFL students in Taiwan appeared to be motivated by requirements rather than clear instrumental yield. Expectancy-value theory and Gardner’s socio-educational model were employed by Mori and Gobel (2006) to investigate Japanese students’ language learning motivation in English as a second language. Ma et al. (2018) examined how intrinsic value among Chinese EFL students promotes their English learning via the mediation effect of self-efficacy beliefs. Razavipour et al. (2021) explored how test takers’ goal mediate test preparation practices for the English module of the higher education admission test. Thus far, relevant empirical studies (e.g., Sun, 2013; Xie and Andrews, 2013; Xie, 2015; Xu, 2017) have applied structural equation modeling to build a model of test preparation in the assessment context of the CET in China. As noted by Sun (2013), learners’ perceptions of the test design and test use directly influenced test preparation, while test-taking expectation worked as the mediating variable between perceptions and test preparation practices. Xie and Andrews (2013) concluded that task value and expectation functioned as different degrees of mediation of the test use and test design and that these constructs worked together to affect test preparation. Xu (2017) focused on test-taking motivation in the CET listening test and concluded that the path from expectancy to test performance was medicated by metacognitive awareness. The available empirical studies concerning the path from perceptions to practices in assessment contexts from expectancy-value perspective triggered interest in this particular research context. Expectancy-value theory posits that individuals’ perceptions of the task demand and goal influence the achievement-related choices by mediating task value and expectation of success. The exact path and effect size in specific research contexts nevertheless call for further exploration.

The vast majority of the studies reviewed above investigated the overall test preparation for various high-stakes tests, while a number of studies focused on test preparation for writing tasks or writing module. Brown (1998) reported that intense test preparation may benefit the International English Language Testing System (IELTS) candidates in terms of successful test performance. Green (2005) explored candidates’ expectations of scores gains on the IELTS academic writing test and offered test preparation recommendations. Furthermore, Green (2007) proposed a model that embodied the features of test design and the characteristics of the IELTS test takers and called for further research with particular reference to establish the evidential links among test takers’ understanding of test demand, their learning goals and their learning behaviors. In China, two studies reported on test preparation for writing component of the National Matriculation English Test (NMET). Qi (2007) observed test preparation activities toward the NMET writing tasks. In her study, senior high school students tended to neglect the writing task demand while emphasizing test preparation strategies and the testing situation. Xu and Wu (2012) analyzed test-taking strategies of 12 high school students using think-aloud and retrospective interview protocols. Driven by the high stakes of the NMET, students developed a whole set of test-taking strategies in preparation process. In sum, the interpretation of specific nature of high-stakes writing tests remains limited. There is a dearth of empirical literature on test preparation for writing tasks from the perspective of expectancy-value theory. In this regard, the paths from perceptions to test-driven preparation practices of the writing tasks need to be further scrutinized. What is of great interest here is to investigate how such effects, both directly and indirectly, come to be and to interpret test preparation mechanism of the GSEEE writing tasks.

## Context of the Study

The higher education system in China is highly selective and competitive. The number of candidates applying for Master’s programs hit a record high of 4.57 million in 2022, with the enrolment rate of approximately 26.8% over the past 3 years. The National Graduate School Entrance Test battery screens candidates for Master’s programs in educational and research institutions (He, 2010). The test battery, with a full score of 500 marks, is comprised of four tests: The politics test (100 marks), the foreign language test (100 marks), and two sub-tests (150 marks each) in subject areas. Administered by the National Education Examinations Authority under the Ministry of Education, the politics test and the foreign language test are two compulsory subjects in the overall testing regime. The foreign language test is available in five different languages (English, Japanese, Russian, French, and German), and more than 90% of the candidates sit for the English module.

Examinations permeate the educational system in China, with language tests playing a vital role. The GSEEE is one of the two large-scale high-stakes English test for admissions purposes, and the other being the NMET (He, 2010). The GSEEE serves dual purposes: To measure test takers’ knowledge of English and their ability to use the language for research; and to offer information for graduate institutes in selecting potential candidates for their master’s programs (He, 2010). The cut-off scores of the GSEEE magnifies its test stakes. There are two cut-off scores, one for the total score of the test battery and the other for the scores on the sub-tests (GSEEE included). Test takers need to score above the cut-off scores in order to be admitted into the Master’s programs in educational and research institutions. Consequently, the stakes associated with the GSEEE are high. Because of the annual testing population (over 1 million from 2005 onward) and the cut-off score, the GSEEE has serious consequences for individual test takers as well as big effects on the educational system.

The GSEEE is a 3-h test with three sections: Use of English, reading comprehension, and two writing tasks. The National Education Examinations Authority released the results of the GSEEE item analyses each year. The mean of the GSEEE test score from 2019 to 2021 was between 47.04 and 49.15; test difficulty between 0.47 and 0.49; and test reliability between 0.81 and 0.85 (NEEA, 2021). The main focus of the present study is the writing section. In practical writing, test takers are required to write memos, reports, and business or personal letters of at least 100 words. Essay writing requires test takers to write an argumentative essay based on the prompt (picture, table, writing outline, etc.) in approximately 160–200 words. All scripts for the GSEEE compositions are double-rated on a holistic scale by accredited raters. Test difficulty of practical writing from 2019 to 2021 was between 0.49 and 0.57, and test difficulty of essay writing from 2019 to 2021 ranges from 0.51 to 0.53 (NEEA, 2021). Of the specific language skills being tested in the GSEEE, writing plays an important role in that it accounts for 30% of the total score. For most Chinese learners of English, writing is an important area of concern as reflected in their lower mean band score compared to the mean band score worldwide (5.5 vs. 5.7 out of 9) in the IELTS (Test Taker Performance, 2019).

Compared with the NMET and the CET, the GSEEE has received relatively little research attention in large-scale assessment studies. He (2010) took an initial step to review the GSEEE in a systemic manner and called for future test validation and washback studies. Research evidence with regard to test fairness of the GSEEE (Song and He, 2015; Song, 2018; Min and He, 2020), the application of the measurement model to the GSEEE reading tasks (Min and He, 2014) and effectiveness of test preparation (Xu, 2021) has also begun to emerge. Nevertheless, the GSEEE remains an under-investigated area that requires more scholarly attention. The present study seeks to quantify the effect of motivational and cognitive perceptions on the test-driven preparation practices of the GSEEE test takers. Two research questions were proposed:

1. What characterizes Chinese EFL test takers’ test preparation practices for the GSEEE writing tasks from an expectancy-value theory perspective?
2. To what extent do motivational and cognitive perceptions affect test-driven preparation practices?

## Materials and Methods

### Participants

The Chinese version of test takers’ questionnaires were distributed to Year 4 undergraduates among 11 universities in a large city in eastern China. Undergraduate students on their test preparation for the GSEEE were recruited voluntarily and were asked to respond to those items in a self-reported manner. Consent forms were obtained from all the participants. Data were collected 1 month before they took the GSEEE. In total, 853 paper version of test takers’ questionnaires were distributed, and 623 valid questionnaires were returned. Table 1 gives the GSEEE test-takers’ profiles in the questionnaire survey. The participants were in their early 20s (*M* = 21.95, *SD* = 1.06) and were relatively balanced by gender, with 327 women and 296 men. They majored in 12 disciplines in their undergraduate studies. Engineering accounted for the largest proportion (44.17%), followed by literature (11.33%), management (10.17%), art (7.83%), and medical science (7.83%). The participants had been learning English for at least 10 years (*M* = 10.83, *SD* = 2.04). The participants were also asked to report their test scores of CET Band 6 (*M* = 473.89 on a 0–750 scale, *SD* = 55.16). The English proficiency level of participants in the present study approximates to B1 level according to the Common European Framework of Reference (Huang and Jia, 2012).

**TABLE 1:** Test-takers’ profiles in the questionnaire survey (*n* = 623).

Institution	Type of the institution	*N*	Percentage
University A	Comprehensive	250	40.13%
University B	Engineering	49	7.87%
University C	Engineering	44	7.06%
University D	Economics	43	6.90%
University E	Sciences	15	2.41%
University F	Sciences	46	7.38%
University G	Comprehensive	44	7.06%
University H	Comprehensive	40	6.42%
University I	Normal	37	5.94%
University J	Arts	37	5.94%
University K	Engineering	18	2.89%
Total		623	100.00%

### Instruments

The perception and practice of the GSEEE test takers’ questionnaire were used in this study. The items regarding perceptions were designed on a 5-point Likert scale of agreement ranging from 1 (totally disagree) to 5 (totally agree), and items related to preparation activities were measured on a 5-point Likert scale ranging from 1 (never were used) to 5 (always) in terms of frequency. The students answered the Chinese version of questionnaires, most of whom completed the questionnaires within around 15–20 min.

Qualitative input and piloting procedures are crucial to ensuring the content and construct validity of a questionnaire survey. The questionnaire was developed through a three-phase process: item generating, initial piloting, and psychometric evaluation. The present study first consulted relevant empirical studies of expectancy-value theory (e.g., Conley, 2012; Xie and Andrews, 2013), relevant literature of test preparation (e.g., Green, 2007; Yu et al., 2017; Razavipour et al., 2021) and scoring criteria for the GSEEE writing tasks (National Education Examinations Authority, 2021). In the second step, 11 participants were invited in a focus-group discussion to describe their perception and preparation activities while preparing for the GSEEE writing tasks. Their responses and comments were used as the item pool for designing the questionnaire survey. Several themes emerged from the focus-group discussion: Test takers’ perceived values of the GSEEE, their attitudes toward the GSEEE as a screening test, perceived effectiveness of test preparation on writing, preparation strategies and learning resources for preparing the GSEEE writing tasks. The themes arising from the focus-group discussion were revised into parts of the questionnaire, which helped to enhance the content validity. The questionnaire items in this study were synthetic and selective based on the following two criteria: (a) items identified by more than six participants in the interview, and (b) established instruments for evaluating expectancy-value theory as supported by the research literature. In total, the initial pool was generated with 35 items. After item generating, pilot study was conducted with another sample (*N* = 66). Data collected from the pilot study were analyzed and modified at two levels: first, participants were invited to comment on the wording of the questionnaire and point out the areas that caused confusion. Questionnaire items which caused confusion were rewritten and paraphrased. Second, items with poor psychometric properties were excluded or modified. The time to complete the questionnaire was also estimated in the pilot study.

In the main study, the perception questionnaire consisted of 28 items and measured four major constructs: test takers’ goal (labeled as goal), perception of task demand of the writing tasks (labeled as task demand), task value of the writing tasks (labeled as task value) and expectation of success (labeled as expectation of success). Goal was measured by test takers’ reasons for sitting for the test and engaging in test preparation. It comprised three items (Cronbach’α = 0.667/3 items), e.g., *My main goal is to be better than other candidates in the writing tasks.* Task demand focused on the writing construct of the GSEEE writing tasks. Test takers were requested to indicate their level of agreement with the scoring criteria for the GSEEE writing tasks. Factor analysis detected three subscales: Mechanics and register (Cronbach’α = 0.887/8 items), e.g., *The writing must meet the requirement on length*; Content and organization (Cronbach’α = 0.876/6 items), e.g., *The writing must effectively address the topic*; Vocabulary and language use (Cronbach’α = 0.772/4 items), e.g., *A wide range of vocabulary is important*. The subscale task value was measured by perceived importance of the writing tasks and comprised three items (Cronbach’α = 0.754/3 items), e.g., *Doing well on the writing tasks helps me to pass the GSEEE*. Expectation of success captured individuals’ beliefs regarding their believed outcomes of the upcoming task (Cronbach’α = 0.844/4 items), e.g., *I have confidence in doing well on the GSEEE writing.* Supplementary Appendix 1 lists the detailed items of test takers’ questionnaire, and Supplementary Appendix 2 lists the descriptive statistics of subscales and correlation matrix.

The practice questionnaire in the main study measured the achievement-related choices in assessment context (labeled as test preparation), which were represented by 23 individual preparation activities (see Supplementary Appendix 1). Test preparation comprised five subscales: Memorizing practice (Cronbach’α = 0.874/5 items), e.g., *Memorize essay structure*; Test familiarization (Cronbach’α = 0.779/6 items), e.g., *Become familiar with the scoring criteria for the GSEEE writing*; Comprehensive learning (Cronbach’α = 0.695/4 items), e.g., *Peer review and revise others’ essays*; Skills development (Cronbach’α = 0.721/5 items), e.g., *Use details and examples to illustrate ideas*; Drilling practice (Cronbach’α = 0.699/3 items) e.g., *Practice simulated tests*.

### Data Analysis

All items were entered into SPSS for preliminary data analysis. Missing responses, normality, and homogeneity for multivariate analyses were checked. The data met the normality assumption of multivariate analysis and were ready to be analyzed. Item-level exploratory factor analysis was carried out first to check whether the items loaded on the theorized constructs. Based on the factors derived from exploratory factor analysis, composite variables were computed by averaging item scores within each factor. Composite variables were used as observable variables in confirmatory factor analysis. Descriptive statistics for observed variables in confirmatory factor analysis are shown in Table 2.

**TABLE 2:** Descriptive statistics for observed variables in structural models.

Variables	*N*	Mean	*SD*	Skewness	Kurtosis
Goal	623	4.12	0.755	–0.674	–0.158
Task value	623	4.19	0.722	–0.872	0.657
Mechanics and register	623	3.32	0.852	–0.256	–0.095
Content and organization	623	3.58	0.836	–0.523	0.206
Vocabulary and language use	623	3.5	0.749	–0.217	0.318
Expectation of success	623	3.64	0.539	–0.398	0.58
Memorizing practice	623	3.76	0.964	–0.598	–0.13
Test familiarization	623	3.2	0.754	0.155	0.256
Comprehensive learning	623	2.29	0.882	0.542	–0.237
Skills development	623	3.37	0.808	–0.188	–0.142
Drilling practice	623	3.51	0.959	–0.377	–0.367

In response to the first research question, structural equation modeling (SEM) was employed to reveal the direct and indirect relations between observable variables and proposed constructs represented by the latent variables (Kline, 2011). AMOS 20 was conducted to unveil the structural relations in the current expectancy-value model and establish the paths from perceptions to preparation practices (Arbuckle, 2006). The ratio of chi-square to its degree of freedom (χ^2^/df), the root mean square error of approximation (RMSEA), the comparative fit index (CFI), the goodness of fit index (GFI), and the Tucker-Lewis index (TLI) were used to measure the overall goodness-of-fit of the model. Specifically, an adequate model fit between the hypothesized model and the observed data should meet the following standard: χ^2^/df < 5, RMSEA < 0.08, CFI > 0.90, GFI > 0.90 and TLI > 0.90 (Kline, 2011). In terms of model parsimony, lower values of Akaike information criteria (AIC) and consistent Akaike information criteria (CAIC) were preferable (Hu and Bentler, 1995).

To proceed the mediation analysis of the second research question, mediation model was constructed, which is widely used in testing theories regarding process mechanism (Preacher and Kelley, 2011). In the mediation model, variable X is postulated to exert an effect on an outcome variable Y through one or more intervening variables, called mediator (M). In the model, *a* refers to the coefficient for X predicting M from X, and *b* and *c′* refer to the coefficients predicting Y from M and X, respectively. In other words, *c′* quantifies the direct effect of X, whereas *a* and *b* quantify the indirect effect of X on Y through M (Hayes, 2009). A bootstrap test was used to check the significance of the indirect effect (Preacher and Hayes, 2004). There should be only one condition to establish mediation, that the indirect effect *a* × *b* be significant (Zhao et al., 2010). Three quantitative measures of relative magnitude were proposed for the estimation of mediating effects (Preacher and Kelley, 2011): *ab* was used to indicate the unstandardized measure of indirect effect whereas β_a_*β_*b*_ referred to the standardized measure of indirect effect. The mediating ratio was defined as *ab/(ab+c′)*, which represented the proportion of the total effect that was mediated.

## Results

### Testing the Competing Models From Expectancy-Value Theory Perspective

**FIGURE 2:**
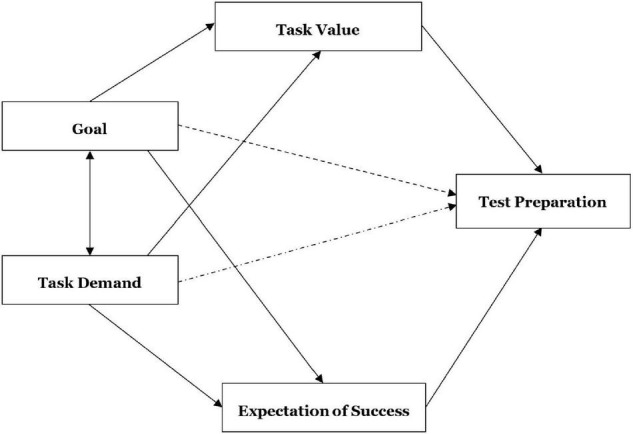
Theoretical models 1–4.

The application of the expectancy-value theory to a real-world situation allowed researchers to test theoretically derived hypotheses, explore the relations among the key constructs and compare various competing models. Specifically, three motivational beliefs (i.e., goal, task value, expectation of success), one cognitive perception (i.e., task demand) and different types of preparation activities were selected in the GSEEE assessment context. Different theoretical models, with slight differences in the paths from perceptions to practices, were specified and presented for statistic modeling: Model 1 (see Figure 2 with all solid lines) was consistent with the original expectancy-value model and hypothesized that goal and task demand influenced test preparation by the two mediating variables (task value and expectation of success). Model 2 (adding the dashed line to Model 1) adds the direct path from goal to test preparation, Model 3 (adding the dashed dotted line to Model 1) added the direct path from task demand to test preparation, and Model 4 (adding the dashed and dashed dotted lines to Model 1) added the direct paths from both goal and task demand to test preparation.

**FIGURE 3:**
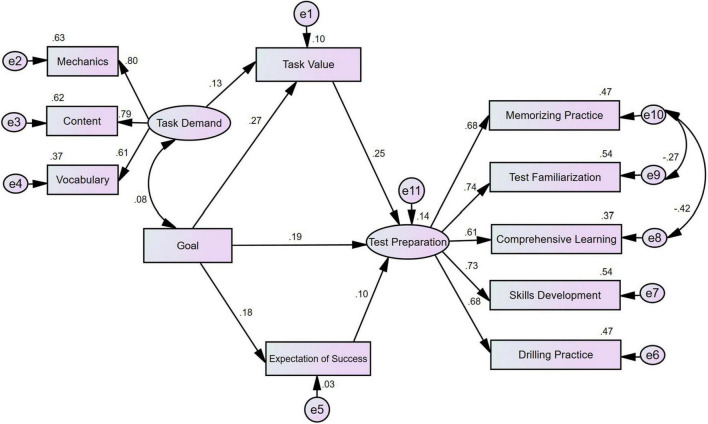
Final model with standardized parameter estimates.

First, four competing models were proposed and tested based on the theoretical model. The four models examine the hypotheses of different paths from perceptions to practices in turn, and the results of the overall model fit were shown in Table 3. The appropriate indices for SEM were set at χ^2^/df < 5, RMSEA < 0.08, CFI > 0.90, GFI > 0.90 and TLI > 0.90 (Kline, 2011). In terms of model parsimony, lower values of AIC and CAIC were preferable (Hu and Bentler, 1995). As shown by the values of fit indices in Table 3, the value of TLI of Model 3 was relatively low, suggesting an inadequate model fit between the hypothesized model and the observed data in Model 3. Thus, Model 3 was excluded from model comparison first. Compared with Model 1 and Model 4, Model 2 demonstrated better values in all the eight fit indices, with lower values of χ^2^/df, RMSEA, AIC and CAIC and higher values of CFI, GFI, and TLI. Therefore, Model 2 was selected as the best-fitting model approximating the perception and practice data in the present study. In Model 2, one path was not significant, i.e., the path from task demand to expectation of success with low regression weight of 0.02 (*p* > 0.05) and was deleted for model parsimony. In the second step, *post hoc* fitting procedures were conducted based on the analyses of the modification indices and theoretical support. Balancing various test preparation practices is a trade-off when test stakes are high (Smith, 1991). In the present study, the memorizing practice was negatively related to comprehensive learning and test familiarization. Empirical studies (e.g., Razavipour et al., 2021; Xu, 2021) have indicated that, due to limited time and energy, preparation activities among test takers might be mutually exclusive in terms of time allotment and preparation patterns. Accordingly, the final model relaxed two constraints by estimating the error covariance associated with memorizing practice (e10) and comprehensive learning (e8) as well as between memorizing practice (e10) and test familiarization (e9). The final model (χ^2^/*df* = 3.239, RMSEA = 0.06, CFI = 0.950, GFI = 0.966, TLI = 0.927, AIC = 179.072, CAIC = 331.239) with its fit indices and path diagram is presented in Figure 3. All of the standardized estimates on the arrow lines were statistically significant at the 0.01 level.

**TABLE 3:** Fit indices of competing models 1, 2, 3, and 4.

	χ^2^/df	RMSEA	CFI	GFI	TLI	AIC	CAIC
Model 1	4.082	0.070	0.929	0.957	0.900	213.179	359.911
Model 2	3.687	0.066	0.940	0.962	0.912	196.115	348.282
Model 3	4.161	0.071	0.929	0.957	0.897	214.129	366.296
Model 4	3.766	0.067	0.939	0.962	0.910	197.349	354.951

*df, degree of freedom; RMSEA, root mean square error of approximation; CFI, comparative fit index; GFI, goodness of fit index; TLI, Tucker-Lewis index; AIC, Akaike information criteria; CAIC, consistent Akaike information criteria.*

As Figure 3 indicates, the latent variable *task demand* was represented and measured by three observed variables: mechanics and register, content and organization, and vocabulary and language use. The factor loadings for task demand, significant at the 0.001 level, ranged from 0.61 to 0.80. Task demand represented test takers’ understanding of the construct of the GSEEE writing tasks, which related closely to scoring criteria for the GSEEE writing tasks. The more extensive the knowledge test takers possessed regarding the writing tasks, the deeper their understanding of the writing construct were assessed by the GSEEE writing. The latent variable *test preparation* loaded on five observed variables: memorizing practice, test familiarization, comprehensive learning, skills development, and drilling practice. The five path loadings were statistically significant at the 0.001 level. The factor loadings for test preparation for the GSEEE writing tasks ranged from 0.61 to 0.74. The latent variable *test preparation* indicated the level of engagement in test preparation practices. The higher level of engagement in test preparation, the more frequently preparation activities were conducted by the GSEEE test takers.

On the whole, expectancy-value theory holds well for the path from test takers’ perceptions to practices of the GSEEE writing tasks. Figure 3 indicates that both goal and task demand positively influenced the GSEEE-driven preparation practices. Test takers who had strong motivations toward the test were apt to conduct various preparation activities more frequently. The total effect of goal on test preparation is moderate (standardized total effect = 0.278). Comprehensive understanding of the task demand of the GSEEE writing tended to facilitate high levels of engagement in preparation practices, though the total effect is slim (standardized total effect = 0.05). Within the competitive higher educational context in the present study, the motivational variable goal exerted more powerful influences on test preparation than the cognitive variable task demand (standardized total effect: 0.278 vs. 0.05).

### Paths of Influence From Perceptions to Test-Driven Preparation Practices

The mediation models constructed in this study involve two mediators (i.e., expectation of success and task value) to explain the effect of motivational and cognitive perceptions to test-driven preparation practices. First, goal functioned as a powerful motivational tool that strongly influenced test preparation practices, similar to the strong effect of required motivation on learning reported by Chen et al. (2005). In this research context, the influence of goal on test preparation was relatively complex. Goal was significantly related to the mediating variables of both task value and expectation of success (β = 0.27, *p* < 0.001 and β = 0.18, *p* < 0.001). In addition to the mediating effect, goal demonstrated a significant positive relation to test preparation adjusted by the two intervening variables (β = 0.19, *p* < 0.001). The slope linking goal to test preparation, controlling for two intervening variables, was significantly different from zero (c′_*goal*–*prep*_ = 0.16, *p* < 0.001). Therefore, the influence of goal on test preparation was partially mediated by task value and expectation of success. Table 4 displays the relative magnitude of the mediating effects of the two paths from goal to preparation practices.

**TABLE 4:** Measurement of mediating effects.

Paths	ab	ab/(ab+c′)	βa*βb
Goal → Test preparation via Task value	0.06	0.272	0.07
Goal → Test preparation via Expectation of success	0.02	0.089	0.02

As indicated in Table 4, intervening variable task value explained a stronger mediating effect on test preparation than the intervening variable expectation of success. In terms of the mediating effect of task value, test takers who had stronger motivations toward the test were likely to attach greater value to the importance of the GSEEE writing tasks. The high endorsed task value as perceived by the test takers then facilitated deep levels of engagement in preparation practices. In the context of colleges/universities, the relation between task values and self-regulated learning across different studies fell into the range of 0.03–0.67 (Pintrich, 1999, p. 463). The present model identified a positive link between task value and test preparation (β = 0.25, *p* < 0.001), controlling for task demand and goal, which suggested a moderate effect of task value in this particular assessment context. In terms of the mediating effect of expectation of success, test takers’ confidence in doing well on the upcoming task was positively related to the level of engagement in preparation practices. Test takers who felt more confident regarding the upcoming task tended to report that they were engaged in more preparation activities in terms of frequency and variety. The present model identified a positive relation between expectation of success and test preparation, controlling for goal (β = 0.10, *p* = 0.013), which deviated slightly from the range of 0.12 to 0.58 as indicated by Pintrich (1999) and suggested a relatively small effect of self-efficacy on test preparation for the GSEEE writing tasks.

Second, the path from task demand to test preparation was relatively straightforward. The influence of task demand on test preparation was completely mediated by the intervening variable task value (c′_*demand*–*prep*_ = 0.04, n.s.), yet the standardized estimates indicated a slim mediating effect (β_*demand*–*value*_ * β_*value*–*prep*_ = 0.03). In other words, test takers’ perceptions and interpretation of the task demand were primarily mediated by the task value although the mediating effect was relatively small when explaining test takers’ engagement in test preparation practices. Washback studies call for empirically evidential links among test design, task demand and the learning behaviors of test takers (Green, 2007). Here, the present model revealed that within this assessment context, test takers’ understanding of the task demand exerted relatively little influence on their GSEEE writing preparation practices. The facilitation of the desired washback effect of tests is based on the assumption that stakeholders adjust their teaching and learning behaviors to the demand of the test. Task demand in the present study, although significant, was insufficient to be termed a strong factor in affecting the preparation practices.

## Discussion

The present study offered empirical evidence of how the expectancy-value model fit into the test-driven preparation practices for the writing tasks of a high-stakes language test in China. The expectancy-value model by Eccles and Wigfield (2002) provides a valuable theoretical framework for the present study. Motivational and cognitive perceptions of the GSEEE test takers, including goal, task value, task demand, and expectation of success were analyzed. Structural equation modeling was conducted to examine the impacts of motivational and cognitive perceptions and the mediating role of task value and expectation of success. The expectancy-value model held well for the paths from perceptions to practices for the GSEEE writing tasks, and the following conclusions were reached: (a) the required motivation (Chen et al., 2005) to take the GSEEE test exerted a strong influence on test preparation practices; (b) task demand, although significant, was inadequate to be termed a strong factor in affecting preparation practices; and (c) task value and expectation of success were mediators from perceptions of goal and task demand to test preparation practices.

The study discussed some enlightening findings. First, the GSEEE test taker’ goal functions as a powerful motivational tool that has huge potential to influence individual’s test preparation practices. In the current higher educational context, test takers’ goal, determined by the high-stakes nature of admission test, exerts a significant influence on the level of engagement in test preparation for GSEEE writing. The results lend support to earlier studies on language learners’ motivation (Chen et al., 2005; Saif et al., 2021). Saif et al. (2021) carried out case studies of test preparation for language tests in Australia, Iran and China and revealed that one commonality across the three contexts is the highly motivated nature of test takers.

The four competing models from expectancy-value theory perspective used in this study was adapted from Xie and Andrews (2013), but the findings are different from theirs. Xie and Andrews (2013), in their study of test preparation mechanism of CET in China, found that the effect of test use (standardized total effect: 0.003) was not as strong as the effects of test design (standardized total effect: 0.386). Considering the context of this study, the possible reasons why the GSEEE test taker’ goal has a major impact are as follows. First, the required motivation to take the GSEEE was much higher than the CET in Xie and Andrew’s study. The minimum English requirement for master’s programs increases the stakes for the GSEEE. For the GSEEE in this study, a single mark below the cut-off score can alter the educational chances of individual test takers. In the CET assessment context, students can sit for the CET twice a year in their 4 years at college (Zheng and Cheng, 2008). In many universities, passing the CET (Band 4 and/or 6) is no longer a part of graduation requirements (Xu, 2017). Second, test preparation in Xie and Andrew’s model was constructed by various types of preparation strategies, the underlying assumption being that the preparation behaviors of the test takers was akin to a rational learning approach whereas test preparation in the present model was defined as the steps, activities and approaches students adopted to prepare for the tests, both desirable and undesirable. Two relevant empirical studies of test preparation for admission English tests also characterize specific test preparation practices in terms of appropriate and inappropriate test preparation activities among test-taking population (Razavipour et al., 2021; Xu, 2021). Razavipour et al. (2021) surveyed test takers in preparing for the English module of the Higher Education Admission Test in Iran and characterized two underlying factors including appropriate and inappropriate preparation patterns. In their study, elven inappropriate test preparation activities were listed, e.g., *memorization is the best way to prepare for the test, if it wasn’t for university entrance exam, I would never have studied English and I can do well on the test without learning the content, just by mastering the test-taking tricks.* Correlation analysis revealed that both mastery and performance goal was significantly related with appropriate preparation activities, but inappropriate preparation activities was significant associated only with mastery goal. Taken together, the incongruence in the available research evidence can be attributed to the distinct construct of test preparation in the respective model. Future studies are recommended to explore the nature and scope of test preparation for high-stakes tests and quantify the relation between goal and test-driven preparation in specific research context.

This study situates its research context in foreign language testing and focuses on writing tasks. The present model reveal that cognitive understanding of the task demand has little influence in determining the preparation process, let alone the learning process. In line with Razavipour et al. (2021), test takers carried out memorizing practice quite intensively in the GSEEE context, e.g., *memorizing all-purpose sentence patterns* and *memorizing essay structure.* The present study was a part of a larger project that investigated the effects of both appropriate and inappropriate test preparation activities on test scores (see Xu, 2021, for more information). After the questionnaire survey, semi-structured interview was carried out among twenty GSEEE test takers. The qualitative data provided supplementary evidence to explain the relatively small effect of task demand on the model. One test taker stated the following:

***S3:*** *As the test day approached, my preparation activities in November had little connection to my previous understanding (of the writing tasks). I just kept on practicing on the past papers and memorizing all-purpose sentence patterns. The same applies to most students. Test preparation cannot be termed a rational process, since most students tend to take the “shortcut” during the intensive preparation period.*

S3 stated clearly that, as the test day approached, his perception of the GSEEE writing tasks had little effect on his preparation practices. S6, who belonged to the group with lower levels of language proficiency, made quite similar comments, indicating that his understanding of the task demand made no difference to his preparation practices. The comments presented above, which serve as supplemental details that describe possible underlying reasons, help to explain the insignificant path from task demand to test preparation in the model.

The findings of this study bear implications on foreign language education and test reform. For test developers, the finding indicates that the overwhelmingly selective function of the GSEEE triggered the required motivation in Chinese EFL students. The strong motivation to excel in the test plays a major role in determining preparation practices, casting a shadow over the cognitive perceptions of the writing tasks to be assessed. Thus, the preparation for the GSEEE writing tasks is not construct-oriented, but rather goal-motivated and stakes-led. The path revealed by the present model responds to Pintrich’s (1999) call for the integration of both motivational and cognitive interventions to promote self-regulated learning and change instructional practices. In high-stakes tests, the test format of multiple-choice items is sometimes blamed, for they tend to result in the coping mechanism and guessing techniques of test-taking experiences (e.g., Benjamin et al., 1984). This study offers empirical evidence to verify the assumption that, when it comes to high-stakes tests, memorizing and drilling practice would apply to any task type and test format, be it multiple-choice items or essay writing (Yang and Gui, 2007).

Nevertheless, the prevailing memorizing practice conducted by the test takers can partly be ascribed to the predictable essay topic and prompt in the GSEEE writing tasks. To promote positive washback, Messick (1996) calls for authentic and direct assessments and minimize construct under-representation and construct-irrelevant difficulty in the test. Given that the GSEEE writing tasks call for relatively short responses (approximately 100 words for practical writing and approximately 160–200 words for essay writing), a lack of authenticity may be to blame for students’ shallow and goal-motivated test preparation. In recent years, National Education Examination Authority in China has been developing a criterion-referenced National English Testing System (NETS), targeting at the scientificity and systematicity of testing as well as the reform and development of foreign language teaching in China (Jiang and He, 2019). The challenge for the GSEEE test reform, is to reframe the test format and further revise the GSEEE writing tasks. Possible direction of further reform may be various task types and formats to eliminate the predictability of essay prompt, which requires more in-depth research of the writing tasks in terms of their similarity to real-world contexts in which graduate students would need to be able to write in English. However, the tension always exists between the general and specific nature of the writing prompt. The challenge for the test designers of the GSEEE writing is, on the one hand, to avoid the potential bias posed by certain specific essay prompt that might be in favor of particular group of test takers, and on the other, to discard those essay topics, prompts and structures that are too general and predictable that can be applied mechanically by certain all-purpose templates prepared by the test takers in advance. The findings of this study suggest that only when test preparation practices are positively related to and significantly influenced by the cognitive demands of the test will the test exert positive washback.

## Conclusion

In the higher education context of China, the present study interprets the test preparation mechanisms of a high-stakes language test from the perspective of expectancy-value theory. As a compulsory test for admission to postgraduate education, the GSEEE is one of the most important English language tests in mainland China. The study focuses on test preparation toward subjective tasks, the GSEEE writing tasks in this case. Our data reveal that the expectancy-value model holds up well for the paths from test takers’ perceptions to test-driven preparation practices, which are not construct-oriented but goal-motivated. Compared with perceptions of task demand, the GSEEE test takers’ goal, determined by the high-stakes nature of admission test, explain their motivation and determine their behavior toward learning, effort and test preparation. Students’ preparation behaviors do not appear to be motivated by specific test features.

In many parts of the world, language tests (English tests in particular) are used for selection and admission purposes. It is necessary to consider the assessment culture and the Chinese EFL setting when investigating the influence of tests on learning motivation. In particular, how test takers perceive high-stakes tests and their personal learning experiences have maximal relevance to the intended washback effect of any high-stakes tests. The research issues presented and discussed here have relevance in many other educational contexts and offer research evidence to enhance the consequential aspect of the construct validity of high-stakes language tests.

Some limitations in this study should be noted along with recommendations for future research. First, given the great educational and social variations in China, 853 test takers involved in data collection cannot fully represent the overall GSEEE test-taking population. A logical extension of the scope of the present study would be longitudinal studies not only to observe potential candidates’ preparation and learning process before the exam but also track their English writing skills and performance in post-graduate studies. How the cognitive and motivational constructs and achievement-related choices relate with one another and change over time merits further investigation. Second, the GSEEE writing section comprises two tasks: practical writing and essay writing. In the questionnaire survey, test takers elaborated on their understanding of each specific task as well as the overall skill they perceive as necessary for fulfilling the writing tasks. It is admitted that two writing tasks tap into different writing constructs and trigger different preparation activities. Future studies may conduct a fine-grained analysis of test preparation mechanism of each specific task and explore the differences. Third, the present study investigates test-driven preparation mechanisms from the expectancy-value theory perspective, which represents an important step in enriching our understanding of washback on learning behavior. However, the model itself is nevertheless inadequate to fully capture the mechanism, and the relatively low standardized estimates suggest that other constructs may be working outside the present model to influence test preparation practices. Given the complexity of human behaviors, more social and motivational factors (e.g., test emotions, test anxiety) and individual differences should be included to extend and complement the present model (Eklöf and Nyroos, 2013; Arendasy et al., 2016; Zhou, 2016). Future empirical studies and extended models are necessary to further explore the fitness of evaluating test preparation mechanism from the expectancy-value theory perspective.

## Data Availability Statement

The raw data supporting the conclusions of this article will be made available by the author, without undue reservation, to any qualified researcher.

## Ethics Statement

Ethical review and approval was not required for the study on human participants in accordance with the local legislation and institutional requirements. The participants provided their written informed consent to participate in this study.

## Author Contributions

The author confirms being the sole contributor of this work and has approved it for publication.

## Conflict of Interest

The author declares that the research was conducted in the absence of any commercial or financial relationships that could be construed as a potential conflict of interest.

## Publisher’s Note

All claims expressed in this article are solely those of the authors and do not necessarily represent those of their affiliated organizations, or those of the publisher, the editors and the reviewers. Any product that may be evaluated in this article, or claim that may be made by its manufacturer, is not guaranteed or endorsed by the publisher.
