# Supplementary material for: Construct-Oriented or Goal-Motivated? Interpreting Test Preparation of a High-Stakes Writing Test From the Perspective of Expectancy-Value Theory
Source: Front Psychol. 2022 Mar 7;13:846413. doi: 10.3389/fpsyg.2022.846413 (PMC8959884; doi:10.3389/fpsyg.2022.846413)
Supplement: Supplementary file 1 [file Table_1.DOC]

Supplementary Material

**Appendix 1**

**Questionnaire for the GSEEE test takers**

| **Goal**  My main goal is to be better than other GSEEE candidates in the writing tasks.  It is important for me to get higher scores than other test takers in the writing tasks.  I want to learn as much as possible from test preparation for the writing tasks.  **Task Demand** |
| --- |
| In order to do well on the test, the writing must effectively address the topic. |
| In order to do well on the test, the writing must include all key points stated in the prompt. |
| In order to do well on the test, proper format of the practical writing is important. |
| In order to do well on the test, the writing must meet the requirement on length. |
| In order to do well on the test, the writing must be well organized |
| In order to do well on the test, grammatical errors are unimportant. |
| In order to do well on the test, appropriate cohesive devices are important. |
| In order to do well on the test, complex sentence structures are important. |
| In order to do well on the test, a wide range of vocabulary is important. |
| In order to do well on the test, appropriate word choice is important. |
| In order to do well on the test, viewpoints must be clearly expressed. |
| In order to do well on the test, graph description must be accurate. |
| In order to do well on the test, the handwriting must be neat and tidy. |
| In order to do well on the test, spelling errors are unimportant. |
| In order to do well on the test, appropriate tone is important. |
| In order to do well on the test, appropriate register is important. |
| In order to do well on the test, logical order of sentences is important. |
| In order to do well on the test, punctuation errors should be avoided. |
| **Task value** |
| Doing well on the writing tasks helps me to pass the GSEEE. |
| Doing well on the GSEEE writing is crucial for the success on the test battery. |
| Doing well on the writing tasks holds the key to high scores of the GSEEE. |
| **Expectation of Success** |
| I have confidence in doing well on the GSEEE writing. |
| I have confidence in passing the GSEEE cut-off score. |
| My preparation for practical writing will boost my writing score. |
| My preparation for essay writing will boost my writing score. |
| **Test Preparation** *(How frequently do you engage in each of the following activities in preparation for the GSEEE writing tasks?)* |
| reading English newspapers and magazines |
| becoming familiar with the essay task |
| becoming familiar with the syllabus of the GSEEE |
| memorizing all-purpose sentence patterns |
| becoming familiar with the scoring criteria for the GSEEE writing |
| getting to know the content areas that the GSEEE writing may cover |
| keeping a diary in English |
| practising on past papers |
| practising simulated tests |
| memorizing the structure of the sample essay |
| becoming familiar with the writing features of different styles and genres |
| using details and examples to illustrate ideas |
| becoming familiar with the practical writing task |
| imitating and rewriting sentences |
| practising writing for a targeted audience |
| memorizing core vocabulary |
| peer review and revising others’ essays |
| completing the writing tasks within the time limit |
| memorizing the template for practical writing |
| memorizing the template for essays |
| revising one’s own composition (content, structure, format, etc.) |
| practising the format of practical writing (memos, letters, reports, etc.) |
| practising the use of cohesive devices |

**Appendix 2**

**Scale descriptive statistics and correlation matrix for all observed indicators**

| Scales |  | Sub-scales | N | Item | Cronbach’α | Mean | SD | F1 | F2 | F3 | F4 | F5 | F6 | F7 | F8 | F9 | F10 | F11 |
| --- | --- | --- | --- | --- | --- | --- | --- | --- | --- | --- | --- | --- | --- | --- | --- | --- | --- | --- |
|  |  | Goal | 623 | 3 | .667 | 4.12 | .755 | / |  |  |  |  |  |  |  |  |  |  |
|  |  | Task value | 623 | 3 | .754 | 4.19 | .722 | .281 | / |  |  |  |  |  |  |  |  |  |
| Task  demand |  | Mechanics and register | 623 | 8 | .887 | 3.32 | .852 | .060 | .081 | / |  |  |  |  |  |  |  |  |
|  |  | Content and organization | 623 | 6 | .876 | 3.58 | .836 | .046 | .130 | .631 | / |  |  |  |  |  |  |  |
|  |  | Vocabulary and language use | 623 | 4 | .772 | 3.50 | .749 | .102 | .170 | .484 | .472 | / |  |  |  |  |  |  |
|  |  | Expectation of success | 623 | 4 | .844 | 3.64 | .539 | .181 | .114 | .034 | .012 | .052 | / |  |  |  |  |  |
| Test  Preparation |  | Memorizing practice | 623 | 5 | .874 | 3.76 | .964 | .227 | .254 | .100 | .137 | .235 | .094 | / |  |  |  |  |
|  |  | Test familiarization | 623 | 6 | .779 | 3.20 | .754 | .210 | .247 | -.021 | .058 | .099 | .156 | .359 | / |  |  |  |
|  |  | Comprehensive learning | 623 | 4 | .695 | 2.29 | .882 | .061 | .117 | -.106 | -.110 | -.019 | .062 | .164 | .481 | / |  |  |
|  |  | Skills development | 623 | 5 | .721 | 3.37 | .808 | .201 | .216 | .102 | .101 | .130 | .166 | .521 | .499 | .463 | / |  |
|  |  | Drilling practice | 623 | 3 | .699 | 3.51 | .959 | .254 | .229 | -.018 | .079 | .101 | .087 | .449 | .525 | .400 | .515 | / |
